# Single-cell transcriptomics of bronchoalveolar lavage reveals divergent macrophage subpopulations and trajectories in interstitial lung disease

**DOI:** 10.1371/journal.pone.0347852

**Published:** 2026-04-29

**Authors:** Lai-Ying Zhang, Peter C. Allen, Viviana P. Lutzky, Simon H. Apte, Penny L. Groves, Maxine E. Tan, Tharushi De Silva, Eleanor R. Spenceley, Rachael A. McCloy, Quan H. Nguyen, Joseph E. Powell, Daniel C. Chambers, Brendan J. O’Sullivan

**Affiliations:** 1 Queensland Lung Transplant Service, The Prince Charles Hospital, Brisbane, Australia; 2 Faculty of Medicine, University of Queensland, Brisbane, Australia; 3 Translational Genomics Program, Garvan Institute of Medical Research, Sydney, Australia; 4 Faculty of Medicine and Health, University of New South Wales, Sydney, Australia; 5 Centre of Immunology and Infection Control, Queensland University of Technology, Brisbane, Australia; 6 Garvan-Weizmann Centre for Cellular Genomics, Garvan Institute of Medical Research, Sydney, Australia; 7 UNSW Cellular Genomics Futures Institute, University of New South Wales, Sydney, Australia; 8 Institute for Molecular Bioscience, University of Queensland, Brisbane, Australia; Medical Center - University of Freiburg, GERMANY

## Abstract

**Rationale:**

Interstitial lung diseases (ILDs) encompass a diverse range of fibrotic conditions and contribute to significant respiratory morbidity and mortality. Assessment of the cellular composition of bronchoalveolar lavage (BAL) fluid is an important diagnostic test in people presenting with ILD, but BAL cellularity remains relatively uncharacterized at single-cell resolution.

**Objective:**

To characterize immune cell populations in BAL across different ILDs and investigate the impact of shortened peripheral blood leukocyte telomere length on BAL immune profiles.

**Methods:**

Single-cell RNA sequencing and downstream analysis were performed on BAL samples from 24 male patients with various ILDs, including idiopathic pulmonary fibrosis (IPF), hypersensitivity pneumonitis, sarcoidosis, and silicosis. Both normal-telomere and short-telomere patients were included. Additionally, we integrated our findings with IPF genome-wide association study (GWAS) data.

**Results:**

We identified sixteen distinct cell populations in BAL with notable differences across ILD subtypes. Analysis revealed six monocyte-like macrophage (MLM) subclusters following divergent trajectories: inflammatory *CXCL10*^hi^ MLMs predominated in hypersensitivity pneumonitis, while pro-fibrotic *SPP1*^hi^ MLMs were significantly expanded in IPF. Short-telomere patients showed a trend toward increased proportion of pro-fibrotic *SPP1*^hi^ MLMs, with enhanced expression of fibrotic genes compared to patients with normal telomere length. Integration with genomic data confirmed that *SPP1*^hi^ and *CCL2*^hi^ MLM subclusters harbour cells with the highest IPF disease relevance scores.

**Conclusion:**

BAL-derived transcriptomics reveals distinct myeloid subpopulations across ILD subtypes, with specific populations associated with disease pathogenesis. These findings provide insight into ILD pathogenesis, motivate the development of more sophisticated diagnostic tests using BAL sampling, and highlight specific myeloid subpopulations as potential therapeutic targets.

## Introduction

Interstitial lung diseases (ILD) encompass a less common but broad subset of chronic lung conditions, including idiopathic pulmonary fibrosis (IPF), hypersensitivity pneumonitis (HP), sarcoidosis, and silicosis, in which abnormal accumulation of stromal cells and extracellular matrix thickens and distorts the pulmonary interstitium. Over time, this causes impaired lung compliance, reduced pulmonary gas exchange, and chronic respiratory failure. While pathogenic molecular signatures in ILD have been shown to extend across multiple biological compartments in various transcriptomic studies, such as in lung parenchyma [1–3] and peripheral blood [4], its landscape in bronchoalveolar lavage (BAL) fluid remains relatively unprofiled at single-cell resolution.

While alveolar macrophages are the predominant cell type in healthy BAL, in pulmonary fibrosis, the cell type composition and gene expression of BAL varies depending on the underlying disease. Single-cell RNA sequencing (scRNAseq) of macrophages isolated from the BAL of fibrotic HP patients, for example, has demonstrated the upregulation of pro-inflammatory pathways compared to IPF [5]. Altered gene expression profiles have been identified in T-helper cells and alveolar macrophages in the BAL of patients with pulmonary sarcoidosis [6]. A recent study integrated the scRNAseq gene expression of peripheral blood monocytes with pulmonary macrophages in the BAL of IPF patients. It inferred a trajectory from classical blood monocytes toward *SPP1* + ve pro-fibrotic BAL macrophages [7], implying a blood-lung recruitment model in pulmonary fibrosis. These studies all however have smaller sample sizes and focus on a single ILD; a broader scRNAseq characterization of multiple ILDs simultaneously is currently absent from the literature. Understanding the distinct cellular and molecular mechanisms that underpin different ILDs has important clinical implications, as these differences may inform diagnostic approaches, guide selection of disease-specific therapies such as antifibrotics versus immunosuppression, and provide insights into prognosis.

In this study, we utilize scRNAseq to characterize immune cell populations within the BAL of patients with a variety of ILDs, including IPF, HP, sarcoidosis, and silicosis, with a focus on myeloid cells. Given the emerging association between shortened telomere length and inferior outcomes in ILD, such as more rapid disease progression and lower transplant-free survival [8], we also describe alterations in BAL macrophages within a subgroup of patients with ILD and shortened age-adjusted peripheral blood leukocyte telomere length. Finally, we confirm the importance of these macrophages in fibrosis by integrating BAL scRNAseq with published IPF genome-wide association study (GWAS) data. This study characterizes the molecular mechanisms specific to various ILDs and defines cellular phenotypes at single-cell resolution, providing new insights into pathogenesis and potential therapeutic targets.

## Methods

### Patient recruitment and sample collection

ILD patients were recruited between 2018 and 2020 from a single tertiary ILD centre in Queensland, Australia, with all ILD diagnoses established through gold-standard, expert multidisciplinary discussion in accordance with international guidelines. BAL samples were collected at time of ILD diagnosis from either the right middle lobe or the lingula in accordance with the American Thoracic Society clinical practice guideline, and fresh-frozen in vapor phase for downstream genotyping and sequencing. Demographic and clinical information was obtained retrospectively on the 25^th^ January 2024 from patient records, clinical letters, respiratory function testing, and radiology. Ethics approval was obtained through the Metro North Hospital and Health Services Human Research Ethics Committee under HREC/2023/MNHB/10028. All patients provided written consent for participation in this study, and all patient data was de-identified during and after data collection.

### Patient relative telomere length phenotyping

Relative telomere length was assessed by flow fluorescence in situ hybridisation (Flow-FISH), using the peptide nucleic acid (PNA) Kit/FITC for Flow Cytometry (DAKO, Agilent Technologies, USA), according to the manufacturer’s instructions. The kit uses fluorescein-conjugated PNA probes to detect telomeres in nucleated haematopoietic cells with the fluorescence intensity of the cells directly correlating with telomere length. Briefly, PBMCs were washed in phosphate-buffered saline (PBS) and mixed 1:1 with control cell line (1301). The samples’ DNA were denatured in a heating block adjusted to 82°C for 10 minutes in hybridization solution with or without fluorescently labelled, telomere specific probe and then incubated overnight at room temperature. The following day, samples were washed, and DNA stained before running on a flow cytometer (LSRFortessa, BD Biosciences). The relative telomere length value was calculated as the ratio between the telomere signal of each sample and the control cell line. To determine the 10^th^ centile cut-off, a standard curve was constructed by measuring the relative telomere length in 150 healthy volunteers (75 males and 75 females, aged 18–77 years old).

### BAL preparation for single-cell RNA sequencing

Cryopreserved BAL cells were thawed and viable cells sorted on a BD Influx cell sorter (Becton Dickinson) using propidium iodide into Dulbecco’s PBS + 0.04% bovine serum albumin and retained on ice. Sorted cells were counted and assessed for viability with Trypan Blue using a Countess automated counter (Invitrogen) and then resuspended at a concentration of 800–1,200 cells/µl.

### Genotyping

To demultiplex the pooled single-cell RNA data, DNA was extracted using Qiagen QIAamp DNA mini kit and genotyping performed using the UKB Axiom array at Ramaciotti Centre for Genomics. Imputation was performed using the Michigan Imputation Server, with Minimac4 and the Haplotype Reference Consortium (HRC) panel.

### Single-cell RNA sequencing

Single-cell RNA sequencing was performed using the following methods. Single-cell suspensions for samples pertaining to *BAL_scRNA_sample#* were loaded onto 10X Genomics Single Cell 3′ Chips along with the RT mastermix as per the manufacturer’s protocol for the Chromium Single Cell 3′ Library (v2, PN-120233; 10X Genomics), to generate single-cell gel beads in emulsion. RT was performed using a C1000 Touch Thermal Cycler with a Deep Well Reaction Module (Bio-Rad) as follows: 55°C for 2 h; 85°C for 5 min; hold 4°C. cDNA was recovered and purified with DynaBeads MyOne Silane Beads (catalog no. 37002D; Thermo Fisher Scientific) and SPRIselect beads (catalog no. B23318; Beckman Coulter). Purified cDNA was amplified as follows: 98°C for 3 min; 12 times (98°C for 15 s, 67°C for 20 s, 72°C for 60 s); 72°C for 60 s; hold 4°C. Amplified cDNA was purified using SPRIselect beads and sheared to ∼200 bp with a Covaris S2 instrument using the manufacturer’s recommended parameters. Sequencing libraries were generated with unique sample indices for each sample. Libraries for the respective samples were multiplexed respectively and sequenced on an Illumina NextSeq 500 (NextSeq control software v2.0.2/ Real Time Analysis v2.4.11) using a 150-cycle NextSeq 500/550 High Output Reagent Kit v2 (FC-404–2002; Illumina).

For samples pertaining to RZ7##, single-cell RNA sequencing was performed in similarly as above but according to the 10x Genomics 3’ scRNAseq protocol, v3.1 and sequenced on an Illumina NovaSeq 6000 using a 200-cycle S4 Reagent Kit (Catalog no. 20028313; Illumina). Sequence reads were aligned using 10X Genomics’s CellRanger software to genome reference GRCh38, Gencode release 44, Ensemble 110. CellBender [9] was also used to adjust for residual RNA in each library. Doublet detection was performed using Demuxafy [10] (v3.0.0) containing the doublet detection packages Demuxalot [11], Vireo [12], scDblFinder [13], scds [14], and Solo [15]. Droplets identified as containing two or more cells were labelled doublets if multiple softwares labelled it so, and were excluded from downstream analyses. For demultiplexing pooled scRNA libraries, we additionally used Demuxafy containing the demultiplex packages Demuxalot and Vireo. Cells were assigned to their respective samples based on genotype information. Only cells with consensus sample assignments across the software tools were retained for further analysis.

Scanpy (v1.10.4) was used to preprocess the scRNA libraries. For quality control, we filtered out cells with fewer than 200 detected genes. Mitochondrial, ribosomal, and hemoglobin gene content was calculated as a percentage for each cell using Scanpy’s *calculate_qc_metrics* function. Cells were filtered out if they fell outside 5 mean absolute deviations (MADs) for log total counts, log number of genes by counts or percentage of counts in the top 20 genes. Additionally, cells with more than 3 MADs for percent mitochondrial counts were removed.

We used the single-cell variational inference (scVI [16]) package to correct for batch effects. Raw counts for each cell were corrected for categorical variables such as pool and 10x chemistry (v2/v3) in addition to continuous variables (percentage of mitochondrial or ribosomal counts and total counts). Default parameters were used with early stopping enabled to prevent overfitting. The resulting latent representation was used to generate the Uniform Manifold Approximation and Projection (UMAP) embeddings for visualization.

Cell-type annotation was performed in a two-step process. In the first step, automated cell annotation methods Celltypist [17] and SCimilarity [18] classified cells using reference-based models for lung [1,19–21] and BAL [22]. Secondly, manual annotation was performed using canonical cell type markers as defined in literature [19,23,24] to ensure proper annotation. A list of these markers can be found in S1 Table.

Processed data can be found on https://doi.org/10.5281/zenodo.18626573.

### Data analysis

Downstream data analyses were performed using Seurat [25] (v5). Cell type proportion analysis was performed using propeller [26], with differences in proportion assessed for statistical significance (*p* < *0.05*) using Seurat’s Wilcoxon rank-sum test. Differential gene expression analysis for each cluster was performed using a combination of the *FindAllMarkers* function in Seurat and edgeR [27] (version 4.0.16).

To further investigate macrophage subclusters, alveolar, monocyte-like, and interstitial-like macrophages were subset and re-clustered for further characterization using Seurat’s *FindAllMarkers*. To identify significant pathways activated in the monocyte-like macrophage subsets, Fast Gene Set Enrichment Analysis [28] (FGSEA, version 1.32.4) was used with pathways listed from the MSigDB hallmark gene set collection [29,30]. Trajectory analysis was performed using slingshot [31] (version 2.14.0) to trace the lineages of MLM subsets. Cellular senescence scoring was calculated using a gene set enriched in senescent cells (SenMayo [32]) as input for the *AddModuleScore* function in Seurat.

### Integrating public GWAS data with scRNA-seq

To identify disease-relevant cells, we applied the single-cell disease relevance score model (scDRS [33], v1.0.3) to our scRNA data. GWAS summary statistics from Allen_IPF [34], UKBB_LymphocyteCount, and UKBB_MonocyteCount were used to find gene sets consisting of top genes with the most significant gene-level *p*-values, which were calculated by combining *p*-values of SNPs nearby to the genes using MAGMA [35] (v1.10). The disease relevance score for each cell was calculated using the command-line interface version of scDRS with default parameters. Covariates for adjusting the scores included in the model for each cell were the number of genes and a binary IPF status.

Results were visualized using Python (v3.13.1) with the matplotlib library [36] (v3.10.0). For plotting disease relevance scores, we utilized UMAP embeddings calculated in the preprocessing step. These embeddings were used to visualize both the full dataset and the subset of monocyte-like macrophages, highlighting cells with high disease relevance scores.

All scripts used to perform preprocessing, integration, annotation, and generate figures can be found on our online GitHub repository (https://github.com/powellgenomicslab/ILD-BAL-scRNA-Manuscript-2025/).

## Results

### Patient demographics

BAL samples were obtained from 24 patients with interstitial lung disease (**Table 1**; further detail in **S2 Table**). Ten patients were diagnosed with idiopathic pulmonary fibrosis (IPF), four with sarcoidosis, three with hypersensitivity pneumonitis (HP), three with smoking-related ILD (SR-ILD), two with silicosis, one with non-specific interstitial pneumonia (NSIP), and one with pleuroparenchymal fibroelastosis (PPFE). Patients were all male, predominantly ex-smokers, and predominantly had mild restriction on spirometry with mild-to-moderate impairment of diffusing capacity, with the exception of the single patient with NSIP who had more severe disease. Non-IPF patients were younger than patients diagnosed with IPF or PPFE, consistent with expected demographics [37]. All patients were treatment-naïve at time of BAL (i.e., no immunosuppressants or anti-fibrotic therapies). Eighteen patients had peripheral blood stored at the time of BAL collection, and a relative telomere length was performed for these patients, with a short peripheral blood leukocyte telomere length defined as a length <10^th^ centile adjusted for age.

**Table 1:** Demographics of patients presenting with interstitial lung disease included in this study.

Diagnosis (n)		Age (yrs, SD)	Ever-Smoker (%)	Short-Telomere (%)	FVC at Diagnosis (%pred, SD)	DLCO at Diagnosis (%pred, SD)
IPF						
	IPF (n = 10)	70.5 (8.0)	90	20	82.8 (11.4)	58.5 (14.3)
Non-IPF						
	HP (n = 3)	61.0 (13.5)	0	67	75.7 (2.5)	53.0 (6.0)
	Sarcoidosis (n = 4)	44.0 (11.1)	75	0	97.0 (10.3)	94.5 (12.8)
	SR-ILD (n = 3)	52.0 (10.0)	100	0	83.3 (6.4)	64.0 (7.8)
	Silicosis (n = 2)	23.5 (0.7)	100	100	97.0 (4.2)	77.0 (0)
	NSIP (n = 1)	74.0 (-)	0	100	62.0 (-)	34.0 (-)
	PPFE (n = 1)	70.0 (-)	0	0	73.0 (-)	54.0 (-)

IPF: idiopathic pulmonary fibrosis. HP: hypersensitivity pneumonitis. PPFE: pleuroparenchymal fibroelastosis. NSIP: non-specific interstitial pneumonia. SR-ILD: smoking-related interstitial lung disease. FVC: forced vital capacity. DLCO: diffusion capacity of the lung for carbon monoxide. %pred: percentage of predicted for age/height.

### Single-cell RNA sequencing of BAL fluid reveals myeloid, lymphoid, and epithelial cell populations present in interstitial lung disease

**Fig 1:**
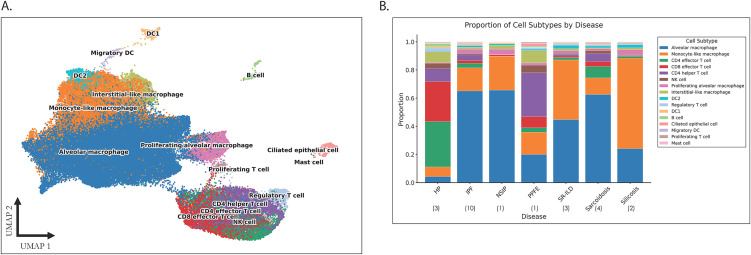
Cell subtypes identified in bronchoalveolar lavage fluid of patients with interstitial lung disease. **A)** Uniform manifold approximation and projection (UMAP) of all cells isolated from bronchoalveolar lavage. **B)** Cell type proportion of each cell subtype stratified by interstitial lung disease subtype. Number of patients pooled within each ILD subtype denoted in brackets. DC: Dendritic cell. ILM: Interstitial-like macrophage. NK: natural killer.

ScRNAseq of BAL from 24 patients with ILD yielded 92,164 individual cells after quality control and filtering. We identified sixteen distinct cell types including macrophages, lymphocytes, dendritic cells (DCs), Mast cells, and a small cluster of ciliated bronchial epithelial cells (**Fig 1a**, **S1 Fig**). Macrophages were further subtyped into alveolar macrophages (AMs), defined by the expression of classic AM markers such as *MARCO*, *PPARG*, and *FABP4*; monocyte-like macrophages (MLMs), expressing both macrophage markers and markers of classical or non-classical monocytes (*VCAN*, *FCN1*, *TYMP*); interstitial-like macrophages (ILMs), expressing markers associated with tissue interstitial macrophages such as *LGMN* and *MARCKS*; and proliferating macrophages. Although previous studies have described monocyte-derived macrophages within BAL [38], analogous to the monocyte-like macrophages we identified, we opted to term them as being “monocyte-like” as it was not possible in our analysis to definitively infer that this population had derived from or differentiated from blood monocytes. Similarly, we describe “interstitial-like” macrophages as this population resembled previously described tissue interstitial perivascular macrophages [19], but were found within the airspace rather than within lung tissue. To compare our macrophage labels, we applied transfer learning to a recent IPF BAL dataset [7]. Our labels accurately mapped to their macrophage cluster, forming distinct groups (**S2 Fig**) that support the validity of our cell-type labels.

Lymphocytes were subtyped into either B cells or T cells, with the latter further subtyped into CD4 and CD8 effector T cells, CD4 helper T cells, regulatory T cells (Tregs), proliferating T cells, or natural killer (NK) cells based on characteristic gene expression profiles. Three subtypes of dendritic cell were identified, including a DC1 population clustered close to macrophages, a DC2 population expressing *CD1E* and *CLEC10A*, and a migratory DC population. Only airway epithelial cells were identified; we identified no alveolar epithelial cells within our analysis.

### Immune cell profiles differ between various ILD subtypes, suggesting divergent mechanisms toward pulmonary fibrosis

Comparison of BAL cell type proportions revealed notable differences between ILD subtypes (Fig 1b, S3 Fig). In IPF patients, alveolar and monocyte-like macrophage populations predominated. In contrast, all three HP patients included in this study had ground-glass nodularity on cross-sectional imaging consistent with an inflammatory HP phenotype [39]; as expected in this context [40], a high proportion of CD4 and CD8 T lymphocytes were identified in BAL via scRNAseq. BAL from sarcoidosis and PPFE patients also revealed an expanded lymphocyte population with an increased CD4:CD8 ratio consistent with published literature [41,42], with a notable prominence of the CD4 helper T cell subtype in PPFE. In contrast, silicosis and SR-ILD BAL demonstrated a significantly increased proportion of monocyte-like macrophages compared to other ILDs, suggesting a role for MLMs in their pathogenesis.

### Transcriptomic analysis of BAL macrophage subpopulations, especially monocyte-like macrophages, reveals distinct phenotypes correlating with ILD subtype

**Fig 2:**
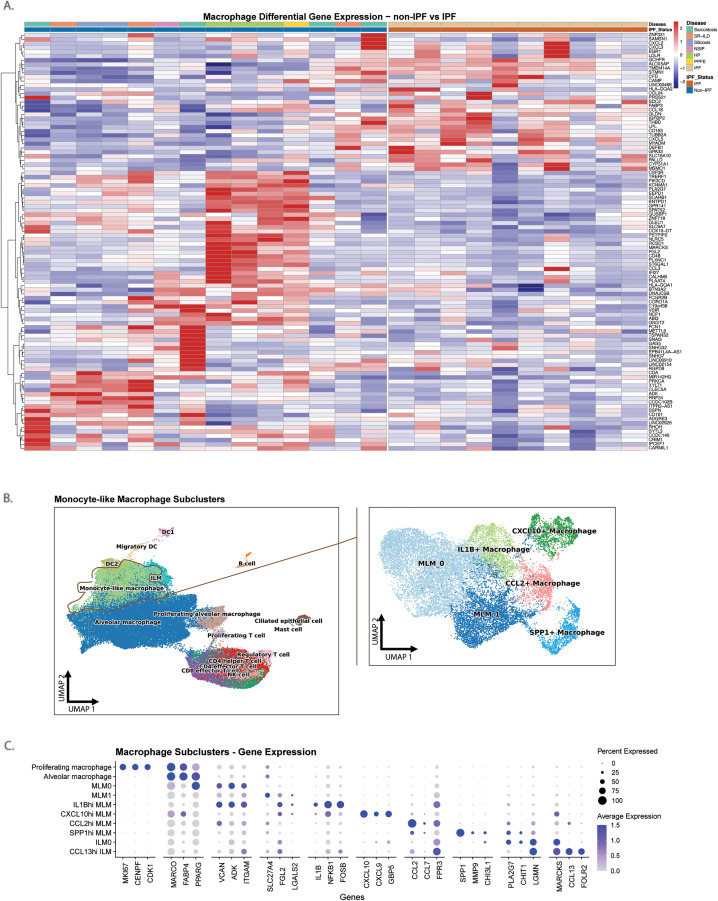
Characterization of macrophage populations in non-IPF and IPF bronchoalveolar lavage. **A)** Heatmap of top differentially expressed genes within BAL macrophages in non-IPF versus IPF patients. **B)** UMAP of monocyte-like macrophage subclusters. **C)** Dotplot of top genes expressed by each macrophage subcluster. MLM: monocyte-like macrophage. ILM: interstitial-like macrophage.

IPF and non-IPF macrophages demonstrated marked transcriptional differences that implied a divergence in macrophage phenotype and function between the two conditions (**Fig 2a**, S3 **Table**). We therefore sought to characterize this further by subclustering the monocyte-like macrophage, alveolar macrophage, and interstitial-like macrophage populations (**Fig 2b**, **2c**).

**Fig 3:**
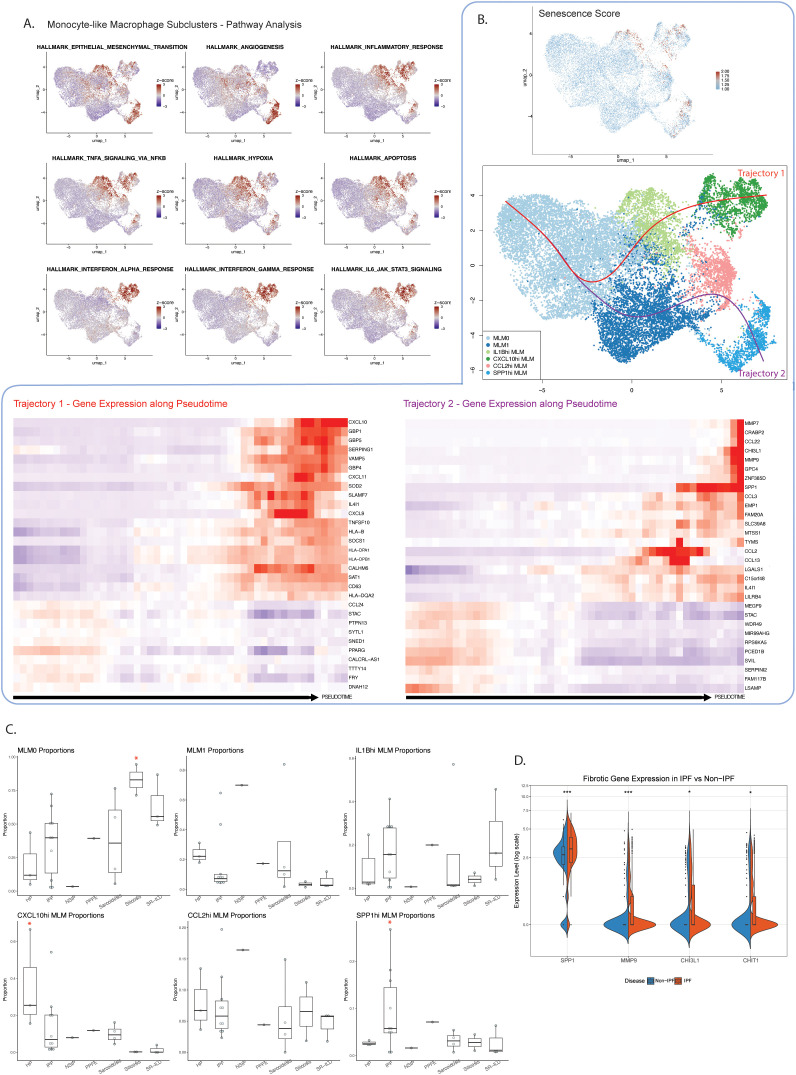
In-depth characterization of BAL monocyte-like macrophages in various ILD subtypes. **A)** MSigDB Hallmark pathway analysis of MLM subclusters, with each highlighted pathway significantly upregulated (adjusted *p* < 0.05) in each subcluster portrayed, as determined by Fast Gene Set Enrichment Analysis. **B)** Trajectory analysis of MLM subclusters demonstrating two differential trajectories, with senescence scoring via SenMayo gene set analysis confirming trajectory directionality. Genes upregulated along each trajectory with progressing pseudotime portrayed via heatmaps. **C)** Cell type proportion analysis of each MLM subcluster (out of all MLMs) in each ILD subtype, with significance (*p* < 0.05) determined via Wilcoxon rank-sum testing. Red *: significantly increased proportion. **D)** Differential expression of selected fibrotic genes in the *SPP1*^hi^ MLM subcluster of non-IPF and IPF monocyte-like macrophages, with significance determined via Wilcoxon rank-sum testing. *: *p* < 0.05, **: *p* < 0.01, ***: *p* < 0.001.

We identified six different monocyte-like macrophage subclusters (Fig 3a, S4A Fig, S4 Table). Importantly, within these subclusters we were able to identify a group of airspace MLMs that upregulated known pro-fibrotic genes such as *SPP1*, *MMP7*, *MMP9*, and *CHI3L1* (denoted as *SPP1*^hi^ MLMs), and which have previously been described within fibrotic lung tissue [1–3]. Pathway analysis revealed that this subcluster was uniquely enriched in pathways associated with epithelial-to-mesenchymal transition (EMT) and angiogenesis, which have previously been implicated in fibrogenesis [1,43]. In contrast, two other MLM subclusters (*IL1B*^hi^ MLMs, *CXCL10*^hi^ MLMs) were found to have gene upregulation patterns associated with pro-inflammatory pathways. *IL1B*^hi^ MLMs demonstrated upregulated *AREG*, *NR4A3*, *SIK1* and other genes associated with canonical TNFα/NFкB signalling, hypoxia, and apoptosis, while *CXCL10*^hi^ MLMs were enriched in IFNα, IFNγ, and the IL6/JAK/STAT3 signalling pathways.

Trajectory analysis was performed to gain insight into possible pathways cells from a specified cluster take when progressing from one cell state to either a terminal state or a more pathogenic state. Of note, a trajectory analysis of MLM subclusters found that pro-fibrotic *SPP1*^hi^ MLMs and inflammatory *CXCL10*^hi^ MLMs existed as terminal endpoints of a divergent trajectory (**Fig 3b**). Cell type proportion analysis found that these two terminal endpoints were differentially expanded in various ILDs; not only was the pro-fibrotic *SPP1*^hi^ MLM cluster significantly expanded in the BAL of patients with IPF in comparison to other ILD subtypes (**Fig 3c**), it also demonstrated significantly upregulated fibrotic gene expression (**Fig 3d**), while *CXCL10*^hi^ MLMs were expanded in the BAL of inflammatory HP patients. When scored for cellular senescence using the SenMayo gene set [32], cells with the highest senescence scores were found toward the terminal endpoints of both MLM trajectories, confirming trajectory directionality. In contrast, silicosis BAL demonstrated a significantly expanded population of early-trajectory MLM0 macrophages.

Subclustering of alveolar macrophages revealed nine subpopulations (**S4b Fig** **and** **S5A**, **S5b Fig**), including a *CCL24*^hi^ AM cluster which upregulated collagen genes (*COL22A1*, *COL23A1*, *COL24A1*), found to be significantly expanded in silicosis BAL (Cluster 3 in **S5C Fig**; *p* = 0.023), and a *PPBP*^*hi*^ AM cluster enriched for various senescence-related genes including *CDK1* and *CCNA2* that was enriched in IPF (Cluster 5 in **S5C Fig**; *p* = 0.0004). We also identified two interstitial-like macrophage subclusters (**S4C Fig** **and** **S6 Fig**) that differentially enriched in inflammatory and adipogenesis pathways. Together, this data highlights the spectrum of phenotypes and functions embodied by various macrophage subsets in fibrotic lung disease.

### Monocyte-like macrophages in short-telomere pulmonary fibrosis BAL may have increased fibrotic potential

**Fig 4:**
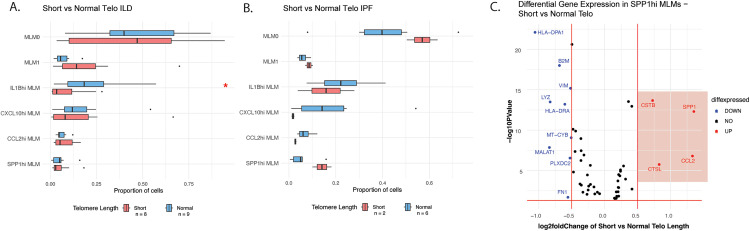
BAL macrophages in short-telomere pulmonary fibrosis are characterized by increased fibrotic potential. Cell type proportion analysis of monocyte-like macrophage subclusters by telomere length, in A) all ILD subtypes (via Wilcoxon rank-sum testing) and **B)** IPF only. *: *p* < 0.05. **C)** Volcano plot of differential gene expression of the *SPP1*^hi^ MLM cluster in short versus normal telomere length IPF, with red genes comparatively upregulated in short-telomere BAL (FDR < 0.05 and log2foldchange > 0.5), and blue genes comparatively downregulated.

Since leukocyte telomere length strongly associates with disease trajectory in ILDs, we next sought to characterize differences in BAL myeloid subpopulations between patients with short and normal age-adjusted peripheral blood leukocyte telomere length. When stratified for telomere length, patients with short-telomere-associated ILD had a reduced proportion of inflammatory MLMs (*IL1B*^hi^ MLMs, *CXCL10*^hi^ MLMs) in comparison with normal-telomere patients (**Fig 4a**). This effect was more prominent when only IPF patients were analyzed, with short-telomere IPF patients having an increased *SPP1*^hi^ MLM population compared to normal-telomere IPF (**Fig 4b**). Furthermore, differential gene expression analysis of the fibrotic *SPP1*^hi^ MLM population comparing short-telomere and normal-telomere IPF patients revealed enhanced expression of *SPP1* and *CCL2* in short-telomere patients, with a relative downregulation of more inflammatory genes such as *MALAT1* and *HLA-DPA1* (**Fig 4c**).

### Integrating GWAS data with scRNA reveals increased IPF-associated signal in SPP1^hi^ and CCL2^hi^ monocyte-like macrophages

**Fig 5:**
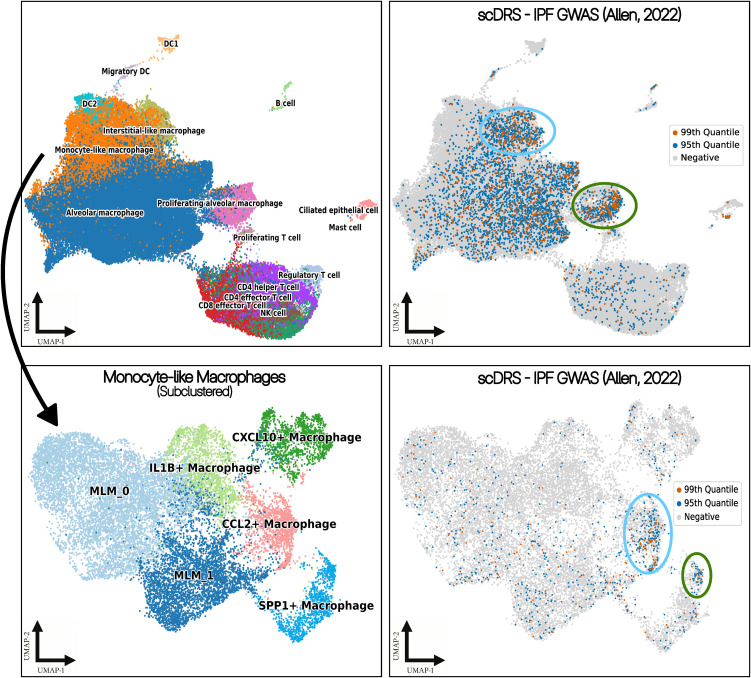
BAL macrophage subpopulations associated with IPF through integration with the Allen *et al.* 2022 genome wide association study (GWAS). Single-cell disease relevance score (scDRS) of each cell to IPF, across all BAL cell types (upper) and in monocyte-like macrophages only (lower), with cells at the 95th percentile in blue and those at the 99th percentile in red. Circles highlight subpopulations with the highest concentration of 95th and 99th percentile scores.

Multiple GWAS to date have identified IPF-associated variants in genes involved in mucin and surfactant production, telomere maintenance, and desmosome function [34,44,45]. Recently, a study linked variants found in these GWAS to gene expression in specific lung cell types through expression quantitative trait loci (eQTL) analysis [46]; however, the sparsity of the data and relatively small sample size makes it challenging to identify true associations between specific cell types and IPF. We applied the single-cell disease relevance score (scDRS) model [33] to our scRNAseq data to identify IPF-relevant cell types in BAL samples to further address this. By intersecting the Allen *et al*. (2022) GWAS with our scRNAseq data, we identified a subset of proliferating alveolar macrophages and monocyte-like macrophages with high disease relevancy scores (**Fig 5**); further subsetting of the monocyte-like macrophages revealed that pro-fibrotic *SPP1*^hi^ and *CCL2*^hi^ MLM subclusters harbored cells with the highest scores. These findings further suggest that these specific macrophage subsets may be crucial to IPF pathogenesis.

## Discussion

Bronchoalveolar lavage is a simple, fast, and safe procedure currently recommended as an important diagnostic tool to differentiate various ILDs based on cell cytology and counts [47]. Unlike surgical or bronchoscopic lung biopsy, BAL is minimally invasive and results in considerably less morbidity, without the risk of triggering a life-threatening ILD exacerbation. The potential utility of BAL-derived cells as a platform for elucidating fibrotic pathophysiological mechanisms, providing insights into disease progression, and identifying biomarkers with prognostic relevance, warrants further investigation. In this study we have successfully sampled and profiled key immune populations within the alveolar space using scRNAseq of BAL cells across a diverse range of ILDs, in patients with both short and normal peripheral blood telomere length. Our findings reveal distinct cellular compositions across different fibrotic conditions, highlighting the heterogeneity of airspace macrophages and their potential contribution to disease pathophysiology.

Previous transcriptomic studies of fibrotic lung tissue have identified a population of pro-fibrotic, monocyte-derived macrophages believed to promote fibrogenesis through interactions with other culprit cells within the fibrotic niche such as aberrant basaloid cells, fibroblasts, and abnormal secretory epithelial cells [48]. In our data, we identify an analogous macrophage subpopulation in BAL. These *SPP1*^hi^ macrophages upregulate similar pro-fibrotic genes to their tissue counterpart, are preferentially enriched in IPF, and particularly in those with telomere shortening. The correlation of our single-cell data with GWAS data confirms a significant association between this *SPP1*^hi^ BAL macrophage subcluster and IPF. Through trajectory analysis, we demonstrate that these macrophages exist in a terminally differentiated state originating from a cluster of macrophages that co-express monocyte markers (MLM0); interestingly, we find that these same MLM0 macrophages can also proceed down a different trajectory toward a more inflammatory terminus (*CXCL10*^hi^ MLMs), especially prominent in HP. In contrast, in silicosis and SR-ILD, both of which show a significant increase in the overall proportion of BAL MLMs, this increase seems to be due to higher proportions of early-trajectory MLM0 macrophages rather than distal-trajectory subclusters.

These findings reveal that lung macrophage populations exhibit remarkable heterogeneity, adopting distinct phenotypes and gene expression profiles – potentially in response to microenvironmental cues or intercellular signaling – suggesting a specialized division of labor among these immune cells. This granular characterization of macrophage subpopulations can provide important insights into immune cell function and behavior that may fundamentally enhance our understanding of ILD disease pathophysiology, or aid in the identification of drug targets. For example, macrophages and monocytes with similar gene expression profiles to the *CXCL10*^hi^ MLMs we describe in inflammatory HP BAL have been previously observed in HP lung tissue [49] and peripheral blood mononuclear cells [7], and are believed to be responsible for CD8 effector T cell chemotaxis into the lung to perpetuate alveolitis [50]. In IPF, conversely, multiple clusters of both alveolar and monocyte-like macrophages in our data are preferentially enriched for fibrotic and senescent, rather than inflammatory, pathways; this effect appears to be most prominent in short-telomere IPF patients, in which there is almost a complete absence of inflammatory *CXCL10*^hi^ MLMs. Clinically, this may partially explain why immunosuppression has been repeatedly demonstrated to be detrimental in IPF and, especially, in short-telomere IPF [51,52], and why short-telomere IPF patients tend to manifest a more aggressive fibrotic phenotype [53].

Additionally, we find that the *SPP1*^hi^ MLM cluster in short-telomere IPF BAL upregulates not only *SPP1*, suggesting increased fibrotic potential, but also upregulates *CCL2*, a chemokine known to increase monocyte and macrophage recruitment [54], in comparison to the analogous cluster in normal-telomere IPF; potentially this might imply a mechanism by which fibrotic macrophages in short-telomere IPF further self-perpetuate fibrosis through a positive feedback loop, recruiting additional monocyte-derived macrophages to the lung to be subsequently directed down a fibrotic trajectory. Elevated levels of *CCL2* have previously been found in the BAL and/or serum of patients with IPF [55], fibrotic HP [49], post-Covid-19 pulmonary fibrosis [56], and connective tissue disease-associated ILD [57], further implying a role in fibrotic progression. While we did not observe a specific variability in the cell type proportion of the *CCL2*^hi^ MLM subcluster between ILDs, this subcluster is present along the fibrotic MLM trajectory. Our analysis of GWAS data found a correlation between IPF and both the *CCL2*^hi^ and *SPP1*^hi^ MLM subclusters, suggesting that both may play an important role in developing and promoting fibrosis.

In silicosis, alveolar macrophages are involved in the phagocytosis of silica particles, which is believed to ultimately trigger apoptosis due to lysosomal disruption [58]; our BAL cell type proportion data correspondingly demonstrates a reduced proportion of AMs and a significantly increased recruitment of likely monocyte-derived macrophages to the lung consistent with published literature in mouse models [59], that are early trajectory and not heavily polarized toward an inflammatory or fibrotic phenotype. As both silicosis patients in our study had early uncomplicated silicosis, the question naturally arises as to whether the early-trajectory MLM0/MLM1 cells seen in their BAL might eventually polarize later in the disease course such as in the development of progressive massive fibrosis, and whether these shifts down particular undesirable trajectories might be capable of modulation or reversal. A recently published study [60] has indeed suggested that airspace macrophages retrieved from patients with silicosis possess significant plasticity and are capable of polarizing toward and away from a pro-fibrotic phenotype when cultured with various drugs *in vitro*, confirming the potential therapeutic relevance of our findings.

Our study presents the first in-depth characterization of BAL macrophage subpopulations across several different ILDs. We provide a detailed description of multiple alveolar, monocyte-like, and interstitial-like macrophage subclusters. We also explore the divergence of monocyte-like macrophages down distinct trajectories into either inflammatory or fibrotic phenotypes depending on the underlying ILD. We are the first to compare, on a single-cell level, BAL from short- and normal-telomere patients with ILD, and demonstrate that the airspace macrophages of short-telomere IPF patients appear to have a greater fibrotic potential.

These findings have several potential implications for ILD management. Our demonstration that BAL-derived scRNAseq can capture disease-relevant macrophage populations analogous to those identified in lung tissue suggests several potential clinical applications. The disease-specific macrophage signatures we identify, particularly the differential enrichment of *SPP1*^hi^ versus *CXCL10*^hi^ MLM subclusters across ILDs, could potentially refine diagnostic approaches in cases where conventional BAL cytology and radiology remain indeterminate. Beyond initial diagnosis, BAL cellular profiling could serve as a platform to inform clinical decision-making in situations where treatment options are unclear or when patients demonstrate unexpected disease progression despite therapy, potentially providing molecular insights into treatment failure or guiding selection between competing therapeutic strategies. The correlation of specific macrophage subclusters with IPF in GWAS data further suggests these populations may have biomarker potential. Additionally, our trajectory analysis identifies a branch point at the MLM0 cluster from which monocyte-like macrophages diverge toward either inflammatory or fibrotic phenotypes; this suggests the existence of a plastic state during which macrophage polarization might potentially be modulated or redirected through therapeutic intervention.

Our study does however have important limitations. Our study cohort is comprised entirely of male patients, which reflects the real-world demographics of patients who underwent BAL during our study period but nevertheless represents an important limitation that restricts the generalizability of our findings. Given the known sex-related differences in ILD prevalence and outcomes [61,62], with male gender especially associated with greater disease progression and worse survival [63], future studies with a more balanced gender representation are needed. Additionally, our study is limited by a small sample size for rarer ILDs, such as NSIP and PPFE (n = 1), which limits robust characterization and comparison for these conditions. We were also only able to analyze two IPF patients with short peripheral blood telomere length which limited our statistical power; a future study with a larger, more balanced cohort of patients would be important to determine whether the findings of our study are generalizable. Furthermore, since our BAL samples were obtained at initial diagnosis, we cannot characterize changes in cell type proportions or phenotypes following disease progression or in response to treatments such as antifibrotics or immunosuppression. It is also important to note that the 10x Genomics technology we utilized is unable to capture neutrophils, which unfortunately limits our understanding of how this cell type contributes to fibrosis. Previous studies have described elevated BAL neutrophil proportions in various ILDs such as IPF [64], SR-ILD [65], and scleroderma-associated ILD [66], with BAL neutrophilia identified as a potential biomarker for reduced survival in progressive ILDs [67] . Indeed, we identified a *PPBP*^*hi*^ alveolar macrophage subcluster enriched in IPF within our study (**S5 Fig**), further implicating BAL neutrophils in fibrosis given *PPBP* is a neutrophil chemoattractant [68]. Therefore, our current study likely does not comprehensively describe the pathophysiology of these conditions and future studies should be employed to more definitively investigate the transcriptome of neutrophils in ILD BAL. Finally, while it was not our study’s objective, we could not directly correlate gene expression or cell type proportions to clinical metrics such as lung function decline over time, which would be important to explore in future studies. It is important to note that our study is exploratory and cross-sectional in nature; we have not directly correlated cellular phenotypes with clinical outcomes such as lung function decline, treatment response, or survival. Whether the macrophage subpopulations and trajectory patterns we describe can reliably predict disease progression, guide treatment selection, or serve as pharmacodynamic biomarkers will require validation in larger prospective cohorts with longitudinal clinical follow-up. Furthermore, the extent to which macrophage phenotypes are modifiable through therapeutic intervention, and whether such modification translates to clinical benefit, remains to be determined.

## Conclusion

Our single-cell RNA sequencing analysis of bronchoalveolar lavage fluid cells reveals distinct cellular compositions across various ILDs, demonstrating that BAL can provide valuable molecular insights through minimally invasive sampling. We were able to characterize diverse macrophage subpopulations and describe airspace monocyte-like macrophages diverging toward inflammatory phenotypes in hypersensitivity pneumonitis, versus fibrotic phenotypes in IPF. Short-telomere IPF patients displayed expanded *SPP1*^hi^ macrophage subpopulations with enhanced fibrotic gene expression, potentially explaining their more aggressive disease course. Integration with GWAS data further highlighted the relevance of specific fibrotic macrophage subclusters in IPF pathogenesis. These findings establish BAL-derived transcriptomics as a powerful approach for understanding fibrotic lung disorders, and suggest specific macrophage subpopulations as potential targets for therapeutic intervention and biomarker development.

## Supporting information

S1 Table Cell type markers used in manual annotation of cell subtypes in BAL. (DOCX)

S2 Table Detailed demographics of patients presenting with interstitial lung disease included in this study. IPF: idiopathic pulmonary fibrosis. HP: hypersensitivity pneumonitis. PPFE: pleuroparenchymal fibroelastosis. NSIP: non-specific interstitial pneumonia. SR-ILD: smoking-related interstitial lung disease. FVC: forced vital capacity. DLCO: diffusion capacity of the lung for carbon monoxide. GORD: gastro-oesophageal reflux disease. %pred: percentage of predicted for age/height.
(DOCX)

S3 Table Differential gene expression between IPF and non-IPF macrophages. Top 20 genes with greatest absolute log2FoldChange between IPF and non-IPF macrophages, with associated adjusted *p*-values included. Positive log2FoldChange indicates relative upregulation in IPF macrophages compared to non-IPF, negative log2FoldChange indicates relative downregulation.
(DOCX)

S4 Table Top 20 marker genes of each monocyte-like macrophage cluster. (DOCX)

S1 Fig Heatmap of top marker gene expression in each cell subtype, in A) myeloid, and B) lymphoid compartments. (TIF)

S2 Fig Label transfer of bronchoalveolar lavage macrophage annotations to an independent dataset of IPF BAL (Zhao *et al*, 2024) demonstrates accurate mapping. (TIF)

S3 Fig Characterization of T and NK cells in fibrotic BAL. Uniform manifold approximation and projection (UMAP) of T and NK cells isolated from ILD BAL, and cell type proportion analysis of each subcluster (out of all T/NK cells) in each ILD subtype. NK: natural killer.
(TIF)

S4 Fig Correlation heatmap plots of gene expression between macrophage subsets in A) monocyte-like macrophages (MLM), B) alveolar macrophages (AM), and C) interstitial-like macrophages (ILM). Red indicates positive correlation, blue indicates negative correlation.
(TIF)

S5 Fig Characterization of alveolar macrophage subclusters in fibrotic BAL. A) Uniform manifold approximation and projection (UMAP) of alveolar macrophage (AM) subclusters isolated from ILD BAL. B) Heatmap of top differentially expressed genes within each AM subcluster. C) Cell type proportion analysis of each alveolar macrophage subcluster (out of all AMs) in each ILD subtype, with significance determined via Wilcoxon rank-sum testing. Only subclusters with significantly different proportions between ILDs are shown. *: *p* < 0.05, **: *p* < 0.01, ***: *p* < 0.001.
(TIF)

S6 Fig Characterization of interstitial-like macrophage subclusters in fibrotic BAL. Uniform manifold approximation and projection (UMAP) of interstitial-like macrophage (ILM) subclusters isolated from ILD BAL, and cell type proportion analysis of each ILM subcluster (out of all ILMs) in each ILD subtype.
(TIF)
