# Supplementary material for: Single-cell transcriptomics of bronchoalveolar lavage reveals divergent macrophage subpopulations and trajectories in interstitial lung disease
Source: PLoS One. 2026 Apr 29;21(4):e0347852. doi: 10.1371/journal.pone.0347852 (PMC13127947; doi:10.1371/journal.pone.0347852)
Supplement: S1 Table — (DOCX) [file pone.0347852.s001.docx]

| Cell Type | Cell Subtype | Gene Markers |
| --- | --- | --- |
| Epithelial |  | *EPCAM* |
|  | Ciliated epithelial | *FOXJ1, TUBB4B* |
| Immune |  | *PTPRC* |
| Immune - Myeloid |  | *TYROBP, LYZ, FCER1G* |
|  | Macrophage | *CD68, GPNMB, CTSB, FN1* |
|  | Alveolar macrophage | *MARCO, PPARG, FABP4* |
|  | Monocyte-like macrophage | *VCAN, FCN1, S100A9, EMP3, TYMP* |
|  | Interstitial-like macrophage | *LGMN, MARCKS, MS4A6A* |
|  | Proliferating macrophage | *STMN1, PTTG1, CKS1B* |
| Immune - Dendritic |  | *ZBTB46, FLT3* |
|  | DC1 | *XCR1, CLEC9A, CLNK* |
|  | DC2 | *PKIB, CLEC10A, CD1E* |
|  | Migratory DC | *TMEM176A, BIRC3, CCL22* |
| Immune - Lymphoid |  | *CD2, GZMA, IL32, NKG7, CD69, CCL5* |
|  | B cell | *MS4A1, BANK1, IGKC* |
|  | NK cell | *NKG7, GNLY, KLRD1* |
|  | T cell | *TRBC2, CD3D, CD3G* |
|  | CD8 effector T cell | *CCL5, CCL4, GZMB* |
|  | CD4 effector T cell | *CD4, CD2, TRAC, IL32* |
|  | CD4 helper T cell | *KLF6, JUND, TRAT1* |
|  | Regulatory T cell | *CCL5 (low), TRAC, IL32* |
| Immune - Mast Cell |  | *MS4A2, SLC18A2, TPSAB1* |
