# Supplementary material for: Single-cell transcriptomics of bronchoalveolar lavage reveals divergent macrophage subpopulations and trajectories in interstitial lung disease
Source: PLoS One. 2026 Apr 29;21(4):e0347852. doi: 10.1371/journal.pone.0347852 (PMC13127947; doi:10.1371/journal.pone.0347852)
Supplement: S2 Table — IPF: idiopathic pulmonary fibrosis. HP: hypersensitivity pneumonitis. PPFE: pleuroparenchymal fibroelastosis. NSIP: non-specific interstitial pneumonia. SR-ILD: smoking-related interstitial lung disease. FVC: forced vital capacity. DLCO: diffusion capacity of the lung for carbon monoxide. GORD: gastro-oesophageal reflux disease. %pred: percentage of predicted for age/height. (DOCX) [file pone.0347852.s002.docx]

| Patient No. | Age | Gender | Ever Smoker | Disease Status | Peripheral Blood Telomere Length | FVC at Diagnosis (L, %pred) | DLCO at Diagnosis (mL/(min*mmHg),  %pred) | Comorbidities |
| --- | --- | --- | --- | --- | --- | --- | --- | --- |
| 1 | 50 | Male | Never | HP | < 10^th^ centile | 4.35 (78%) | 20.09 (59%) | Obesity |
| 2 | 76 | Male | Never | HP | < 10^th^ centile | 3.12 (76%) | 11.66 (47%) | Ischemic heart disease, monoclonal gammopathy of unknown significance, GORD |
| 3 | 57 | Male | Never | HP | < 10^th^ centile | 3.06 (73%) | 14.58 (53%) | Squamous cell carcinoma, GORD, hypothyroidism |
| 4 | 63 | Male | Ex-smoker | IPF | > 10^th^ centile | 3.46 (74%) | 15.00 (53%) | Ischemic heart disease, type 2 diabetes mellitus, hypertension, dyslipidemia |
| 5 | 71 | Male | Ex-smoker | IPF | > 10^th^ centile | 4.10 (78%) | 15.89 (50%) | Ischemic heart disease, osteoarthritis, restless legs syndrome |
| 6 | 70 | Male | Ex-smoker | IPF | > 10^th^ centile | 3.37 (84%) | 13.56 (82%) | Testosterone deficiency, laryngeal carcinoma, GORD, obstructive sleep apnoea |
| 7 | 81 | Male | Ex-smoker | IPF | > 10^th^ centile | 3.26 (93%) | 13.87 (61%) | Hypertension, dyslipidemia, benign prostatic hypertrophy, total knee replacement, benign pulmonary nodule |
| 8 | 66 | Male | Ex-smoker | IPF | > 10^th^ centile | 3.75 (78%) | 22.08 (76%) | Hypertension, GORD |
| 9 | 75 | Male | Never | IPF | < 10^th^ centile | 3.76 (106%) | 13.38 (57%) | Hypertension, osteomyelitis, gout, GORD, dyslipidemia |
| 10 | 78 | Male | Ex-smoker | IPF | > 10^th^ centile | 2.40 (Unk) | 15.20 (Unk) | Ischemic heart disease, type 2 diabetes mellitus, prostate cancer, gout, asthma, chronic kidney disease |
| 11 | 77 | Male | Ex-smoker | IPF | Unknown | 3.21 (75%) | 10.20 (39%) | Hypertension, atrial fibrillation, aortic stenosis |
| 12 | 54 | Male | Ex-smoker | IPF | Unknown | 4.76 (Unk) | 27.79 (Unk) | Gout, GORD |
| 13 | 70 | Male | Ex-smoker | IPF | < 10^th^ centile | 3.41 (74%) | 19.89 (50%) | Ischemic heart disease, idiopathic constrictive pericarditis |
| 14 | 70 | Male | Unknown | PPFE | > 10^th^ centile | 3.45 (73%) | 15.89 (54%) | Ischemic heart disease, right internal iliac aneurysm, squamous cell carcinoma |
| 15 | 74 | Male | Unknown | NSIP | < 10^th^ centile | 2.34 (62%) | 8.10 (34%) | Ischemic heart disease |
| 16 | 45 | Male | Ex-smoker | Sarcoidosis | Unknown | 5.62 (102%) | 34.23 (104%) | Pauci-immune glomerulonephritis, gout, GORD |
| 17 | 59 | Male | Ex-smoker | Sarcoidosis | > 10^th^ centile | 4.07 (82%) | 24.89 (82%) | Allergic rhinitis, dyslipidemia |
| 18 | 39 | Male | Unknown | Sarcoidosis | Unknown | 5.75 (99%) | 38.06 (107%) | Allergic rhinitis, obstructive sleep apnoea |
| 19 | 33 | Male | Ex-smoker | Sarcoidosis | > 10^th^ centile | 5.54 (105%) | 26.29 (85%) | Epilepsy, ankylosing spondylitis |
| 20 | 23 | Male | Current | Silicosis | < 10^th^ centile | 5.20 (94%) | 28.59 (77%) | Allergic rhinitis, childhood asthma, adrenal adenoma |
| 21 | 24 | Male | Ex-smoker | Silicosis | < 10^th^ centile | 5.64 (100%) | 28.80 (77%) | None |
| 22 | 42 | Male | Current | SR-ILD | > 10^th^ centile | 4.12 (87%) | 23.16 (73%) | Epilepsy |
| 23 | 52 | Male | Ex-smoker | SR-ILD | Unknown | 4.47 (87%) | 18.79 (60%) | GORD |
| 24 | 62 | Male | Ex-smoker | SR-ILD | Unknown | 3.54 (76%) | 16.43 (59%) | Dyslipidemia, type 2 diabetes mellitus |
