# Supplementary material for: Single-cell transcriptomics of bronchoalveolar lavage reveals divergent macrophage subpopulations and trajectories in interstitial lung disease
Source: PLoS One. 2026 Apr 29;21(4):e0347852. doi: 10.1371/journal.pone.0347852 (PMC13127947; doi:10.1371/journal.pone.0347852)
Supplement: S3 Table — Top 20 genes with greatest absolute log2FoldChange between IPF and non-IPF macrophages, with associated adjusted p-values included. Positive log2FoldChange indicates relative upregulation in IPF macrophages compared to non-IPF, negative log2FoldChange indicates relative downregulation. (DOCX) [file pone.0347852.s003.docx]

| **Gene** | **Average log2FoldChange** | **Adjusted p value** |
| --- | --- | --- |
| LINC00486 | 2.462104327 | 9.3812012280832e-92 |
| CXCL5 | 2.14124639 | 1.002108179264e-63 |
| IGFBP2 | 1.387507761 | 8.36005968725863e-68 |
| HLA-DQA2 | 1.355721736 | 8.67691613797335e-90 |
| DEFB1 | 1.282127055 | 9.6597555706729e-47 |
| EGR1 | 1.250010357 | 1.29867700764061e-13 |
| SDC2 | 1.142431645 | 4.50290588313581e-16 |
| FABP3 | 1.106888933 | 2.36308095403349e-15 |
| CCL24 | 1.097335718 | 8.56456091412211e-09 |
| GPA33 | 1.042032118 | 9.81219588190272e-34 |
| ITPR2-AS1 | -1.049906978 | 6.22528802238832e-28 |
| CD48 | -1.115740176 | 1.84438179739986e-38 |
| TRERF1 | -1.136401692 | 5.96066427521818e-25 |
| C19orf38 | -1.140024148 | 2.17195934458848e-22 |
| GPR141 | -1.221087306 | 3.80631471762662e-27 |
| ADK | -1.230747797 | 2.47067443263381e-128 |
| ENSG00000226281 | -1.25548485 | 8.91008987127425e-48 |
| CDA | -1.263946995 | 2.64967295712488e-24 |
| ADGRE3 | -1.294071825 | 3.29750846573716e-28 |
| FCN1 | -1.380364176 | 4.25482372218954e-59 |
