# Supplementary material for: Single-cell transcriptomics of bronchoalveolar lavage reveals divergent macrophage subpopulations and trajectories in interstitial lung disease
Source: PLoS One. 2026 Apr 29;21(4):e0347852. doi: 10.1371/journal.pone.0347852 (PMC13127947; doi:10.1371/journal.pone.0347852)
Supplement: S4 Table — (DOCX) [file pone.0347852.s004.docx]

| **Cluster** | **Gene** | **avg_log2FC** | **p_val_adj** | **pct.1** | **pct.2** |
| --- | --- | --- | --- | --- | --- |
| MLM0 | ARHGEF28 | 2.35732781 | 0 | 0.294 | 0.075 |
| MLM0 | THBS1 | 2.22455706 | 5.15508533972949e-289 | 0.284 | 0.079 |
| MLM0 | ANO5 | 2.02969859 | 0 | 0.357 | 0.129 |
| MLM0 | SVIL | 1.94981806 | 0 | 0.833 | 0.492 |
| MLM0 | PPARG | 1.82311229 | 0 | 0.945 | 0.75 |
| MLM0 | PCOLCE2 | 1.81302524 | 8.17058909563816e-215 | 0.272 | 0.095 |
| MLM0 | TSEN2 | 1.79776272 | 3.20518766141296e-199 | 0.286 | 0.113 |
| MLM0 | TCF7L2 | 1.75288232 | 0 | 0.931 | 0.647 |
| MLM0 | PRKN | 1.75058847 | 3.05469504624389e-174 | 0.259 | 0.102 |
| MLM0 | LSAMP | 1.68931704 | 1.41692355164629e-241 | 0.531 | 0.33 |
| MLM0 | DNAAF4 | 1.68769785 | 2.9712912669044e-187 | 0.271 | 0.105 |
| MLM0 | ATP10A | 1.64210585 | 7.86570034898879e-199 | 0.346 | 0.164 |
| MLM0 | PDE3B | 1.6277703 | 0 | 0.522 | 0.262 |
| MLM0 | ENSG00000237356 | 1.62545094 | 2.95705882036421e-170 | 0.277 | 0.117 |
| MLM0 | PEX14 | 1.57835102 | 0 | 0.56 | 0.291 |
| MLM0 | PCED1B | 1.57819082 | 0 | 0.705 | 0.435 |
| MLM0 | ENSG00000234147 | 1.48384425 | 1.46706268296032e-252 | 0.496 | 0.285 |
| MLM0 | TANC2 | 1.48068551 | 0 | 0.839 | 0.618 |
| MLM0 | CORO2A | 1.46812067 | 1.35102318307068e-183 | 0.324 | 0.148 |
| MLM0 | FIG4 | 1.46282334 | 0 | 0.539 | 0.291 |
| CCL2hi MLM | CCL2 | 4.4930449 | 0 | 1 | 0.15 |
| CCL2hi MLM | CCL13 | 4.01178637 | 1.59816001499772e-219 | 0.275 | 0.053 |
| CCL2hi MLM | MERTK | 2.12980783 | 1.01533799855646e-181 | 0.33 | 0.085 |
| CCL2hi MLM | LGMN | 1.71536541 | 5.33585968401585e-118 | 0.461 | 0.213 |
| CCL2hi MLM | CTSL | 1.53668288 | 1.14699937145386e-146 | 0.802 | 0.569 |
| CCL2hi MLM | RNASE1 | 1.3287982 | 6.39368514090961e-72 | 0.393 | 0.193 |
| CCL2hi MLM | PLA2G7 | 1.2979177 | 6.06640205178684e-66 | 0.375 | 0.189 |
| CCL2hi MLM | RGL1 | 1.22636364 | 2.45599261459388e-41 | 0.254 | 0.122 |
| CCL2hi MLM | EMP1 | 1.21790302 | 1.72099143163254e-103 | 0.592 | 0.334 |
| CCL2hi MLM | MARCKS | 1.11149412 | 1.02563018854232e-79 | 0.466 | 0.242 |
| CCL2hi MLM | TIMP1 | 1.10277796 | 5.23888506947462e-40 | 0.658 | 0.547 |
| CCL2hi MLM | PLAU | 1.10270968 | 1.36002170292685e-27 | 0.314 | 0.196 |
| CCL2hi MLM | PMP22 | 1.10200775 | 1.73089399761221e-67 | 0.494 | 0.292 |
| CCL2hi MLM | FMN1 | 1.0736728 | 5.29131402079789e-96 | 0.56 | 0.298 |
| CCL2hi MLM | TMEM163 | 1.03076565 | 3.71629277423448e-64 | 0.353 | 0.165 |
| CCL2hi MLM | MAFB | 1.01304602 | 6.24485143843848e-82 | 0.643 | 0.426 |
| CCL2hi MLM | HMOX1 | 0.99336285 | 0.01027334 | 0.401 | 0.358 |
| CCL2hi MLM | CCL18 | 0.98787366 | 5.52456436800027e-24 | 0.644 | 0.483 |
| CCL2hi MLM | SDF2L1 | 0.98697103 | 1.23366098555874e-28 | 0.407 | 0.283 |
| CCL2hi MLM | TTYH3 | 0.97630264 | 1.91904428161559e-27 | 0.296 | 0.176 |
| MLM1 | SLC27A4 | 1.6569007 | 0 | 0.534 | 0.209 |
| MLM1 | ID3 | 1.45896813 | 1.62781597379368e-121 | 0.261 | 0.116 |
| MLM1 | CLEC10A | 1.43919252 | 7.65675060654268e-141 | 0.294 | 0.129 |
| MLM1 | RPL7 | 1.34383899 | 0 | 0.982 | 0.884 |
| MLM1 | SNHG32 | 1.33918971 | 9.83248755075549e-162 | 0.408 | 0.214 |
| MLM1 | RPL31 | 1.33453147 | 0 | 0.958 | 0.808 |
| MLM1 | RPL13A | 1.32533582 | 0 | 0.998 | 0.978 |
| MLM1 | RPS17 | 1.3200518 | 0 | 0.98 | 0.877 |
| MLM1 | EEF1G | 1.30233316 | 0 | 0.941 | 0.792 |
| MLM1 | HLA-DPB1 | 1.25798931 | 0 | 0.999 | 0.985 |
| MLM1 | MS4A6A | 1.22911887 | 0 | 0.833 | 0.563 |
| MLM1 | SNHG5 | 1.2241952 | 1.16636801199961e-213 | 0.64 | 0.438 |
| MLM1 | NOP53 | 1.1738078 | 1.07412230265844e-161 | 0.503 | 0.312 |
| MLM1 | IDH2 | 1.16970287 | 1.05699940319468e-104 | 0.299 | 0.152 |
| MLM1 | RPS3 | 1.13725614 | 0 | 0.997 | 0.944 |
| MLM1 | TMSB4X | 1.13224059 | 0 | 1 | 1 |
| MLM1 | MIF4GD | 1.12947654 | 6.18971008084534e-81 | 0.256 | 0.134 |
| MLM1 | RPS2 | 1.10081651 | 0 | 1 | 0.992 |
| MLM1 | CORO1A | 1.05007681 | 1.14100396651801e-147 | 0.483 | 0.284 |
| MLM1 | RPS4X | 1.03978252 | 0 | 0.992 | 0.933 |
| IL1Bhi MLM | EREG | 4.43592383 | 0 | 0.405 | 0.068 |
| IL1Bhi MLM | IL1B | 3.62068132 | 0 | 0.437 | 0.097 |
| IL1Bhi MLM | NR4A3 | 3.59038969 | 0 | 0.371 | 0.049 |
| IL1Bhi MLM | PID1 | 3.54722938 | 0 | 0.505 | 0.113 |
| IL1Bhi MLM | EGR1 | 3.27127012 | 0 | 0.492 | 0.1 |
| IL1Bhi MLM | CXCL8 | 3.2711456 | 0 | 0.498 | 0.101 |
| IL1Bhi MLM | NR4A1 | 3.2127962 | 0 | 0.335 | 0.048 |
| IL1Bhi MLM | NLRP3 | 3.18234875 | 5.21018920303955e-251 | 0.306 | 0.065 |
| IL1Bhi MLM | GPR183 | 3.13988463 | 0 | 0.424 | 0.086 |
| IL1Bhi MLM | MAILR | 3.13695355 | 2.24749032335061e-239 | 0.295 | 0.064 |
| IL1Bhi MLM | ZNF331 | 3.08803113 | 0 | 0.584 | 0.184 |
| IL1Bhi MLM | VEGFA | 3.07546569 | 4.08936947117909e-293 | 0.276 | 0.045 |
| IL1Bhi MLM | CXCL2 | 3.02271405 | 1.93372385930714e-234 | 0.375 | 0.108 |
| IL1Bhi MLM | PLEKHG2 | 2.99494039 | 3.65195017008723e-299 | 0.303 | 0.054 |
| IL1Bhi MLM | RGS1 | 2.92233012 | 0 | 0.349 | 0.071 |
| IL1Bhi MLM | SLC7A5 | 2.89342488 | 1.20090897279771e-280 | 0.29 | 0.052 |
| IL1Bhi MLM | NR4A2 | 2.88737063 | 0 | 0.648 | 0.15 |
| IL1Bhi MLM | FOSB | 2.67959326 | 0 | 0.846 | 0.28 |
| IL1Bhi MLM | RGS2 | 2.65087794 | 2.51635210924845e-293 | 0.374 | 0.085 |
| IL1Bhi MLM | NAMPT | 2.61943126 | 0 | 0.893 | 0.471 |
| SPP1hi MLM | SPP1 | 8.83685358 | 0 | 0.929 | 0.047 |
| SPP1hi MLM | CHI3L1 | 6.71219612 | 0 | 0.291 | 0.014 |
| SPP1hi MLM | MMP9 | 6.11120101 | 0 | 0.277 | 0.018 |
| SPP1hi MLM | CHIT1 | 5.87190293 | 0 | 0.289 | 0.02 |
| SPP1hi MLM | TM4SF19 | 4.24652815 | 0 | 0.318 | 0.038 |
| SPP1hi MLM | SDC2 | 3.48010537 | 0 | 0.678 | 0.118 |
| SPP1hi MLM | MATK | 2.89828777 | 5.0151732597032e-264 | 0.438 | 0.103 |
| SPP1hi MLM | PALLD | 2.72963948 | 4.13802211373982e-180 | 0.346 | 0.087 |
| SPP1hi MLM | IL1RN | 2.59105509 | 1.43344833563972e-134 | 0.472 | 0.199 |
| SPP1hi MLM | MREG | 2.32393468 | 3.36497761225284e-125 | 0.259 | 0.065 |
| SPP1hi MLM | MGLL | 2.3104336 | 4.66668431733857e-132 | 0.326 | 0.097 |
| SPP1hi MLM | SPARC | 2.26333169 | 2.94629151330527e-63 | 0.253 | 0.099 |
| SPP1hi MLM | SLC16A10 | 2.16992388 | 5.85614004740364e-167 | 0.368 | 0.1 |
| SPP1hi MLM | FAM20C | 2.07029878 | 1.78752549279246e-163 | 0.426 | 0.136 |
| SPP1hi MLM | LINC01010 | 2.03229398 | 7.66960999963993e-115 | 0.433 | 0.177 |
| SPP1hi MLM | APOE | 1.93763541 | 2.72327615324885e-94 | 0.883 | 0.741 |
| SPP1hi MLM | FABP5 | 1.91229112 | 8.27594726610975e-272 | 0.959 | 0.833 |
| SPP1hi MLM | NR1H3 | 1.89964335 | 8.39883162029517e-85 | 0.289 | 0.103 |
| SPP1hi MLM | CTSL | 1.88975046 | 2.2473720217342e-183 | 0.839 | 0.57 |
| SPP1hi MLM | PLA2G7 | 1.86709875 | 4.23511234424222e-131 | 0.468 | 0.185 |
| CXCL10hi MLM | CXCL10 | 7.5704618 | 0 | 0.859 | 0.035 |
| CXCL10hi MLM | CXCL9 | 6.8421913 | 0 | 0.521 | 0.026 |
| CXCL10hi MLM | CXCL11 | 6.77392911 | 0 | 0.44 | 0.01 |
| CXCL10hi MLM | GBP5 | 4.63869376 | 0 | 0.732 | 0.076 |
| CXCL10hi MLM | APOBEC3A | 4.62551263 | 0 | 0.27 | 0.021 |
| CXCL10hi MLM | ISG20 | 4.20800852 | 0 | 0.304 | 0.026 |
| CXCL10hi MLM | ENSG00000227531 | 4.11976103 | 0 | 0.259 | 0.021 |
| CXCL10hi MLM | GBP1P1 | 4.11478928 | 0 | 0.269 | 0.017 |
| CXCL10hi MLM | TNFAIP6 | 4.09757165 | 0 | 0.262 | 0.026 |
| CXCL10hi MLM | ENSG00000285744 | 4.096296 | 0 | 0.426 | 0.043 |
| CXCL10hi MLM | CCL4 | 3.95642913 | 0 | 0.339 | 0.048 |
| CXCL10hi MLM | GBP4 | 3.87524824 | 0 | 0.706 | 0.098 |
| CXCL10hi MLM | GBP1 | 3.85273728 | 0 | 0.922 | 0.256 |
| CXCL10hi MLM | IFI27 | 3.73971828 | 0 | 0.423 | 0.088 |
| CXCL10hi MLM | RSAD2 | 3.68974946 | 0 | 0.346 | 0.055 |
| CXCL10hi MLM | CCL4L2 | 3.66372857 | 0 | 0.281 | 0.039 |
| CXCL10hi MLM | SLAMF7 | 3.6590068 | 0 | 0.4 | 0.051 |
| CXCL10hi MLM | CD274 | 3.47941023 | 0 | 0.417 | 0.058 |
| CXCL10hi MLM | USP30-AS1 | 3.27981935 | 0 | 0.317 | 0.04 |
| CXCL10hi MLM | SERPING1 | 3.26313128 | 0 | 0.796 | 0.231 |
