# Supplementary figures and images for: Single-cell transcriptomics of bronchoalveolar lavage reveals divergent macrophage subpopulations and trajectories in interstitial lung disease
Source: PLoS One. 2026 Apr 29;21(4):e0347852. doi: 10.1371/journal.pone.0347852 (PMC13127947; doi:10.1371/journal.pone.0347852)

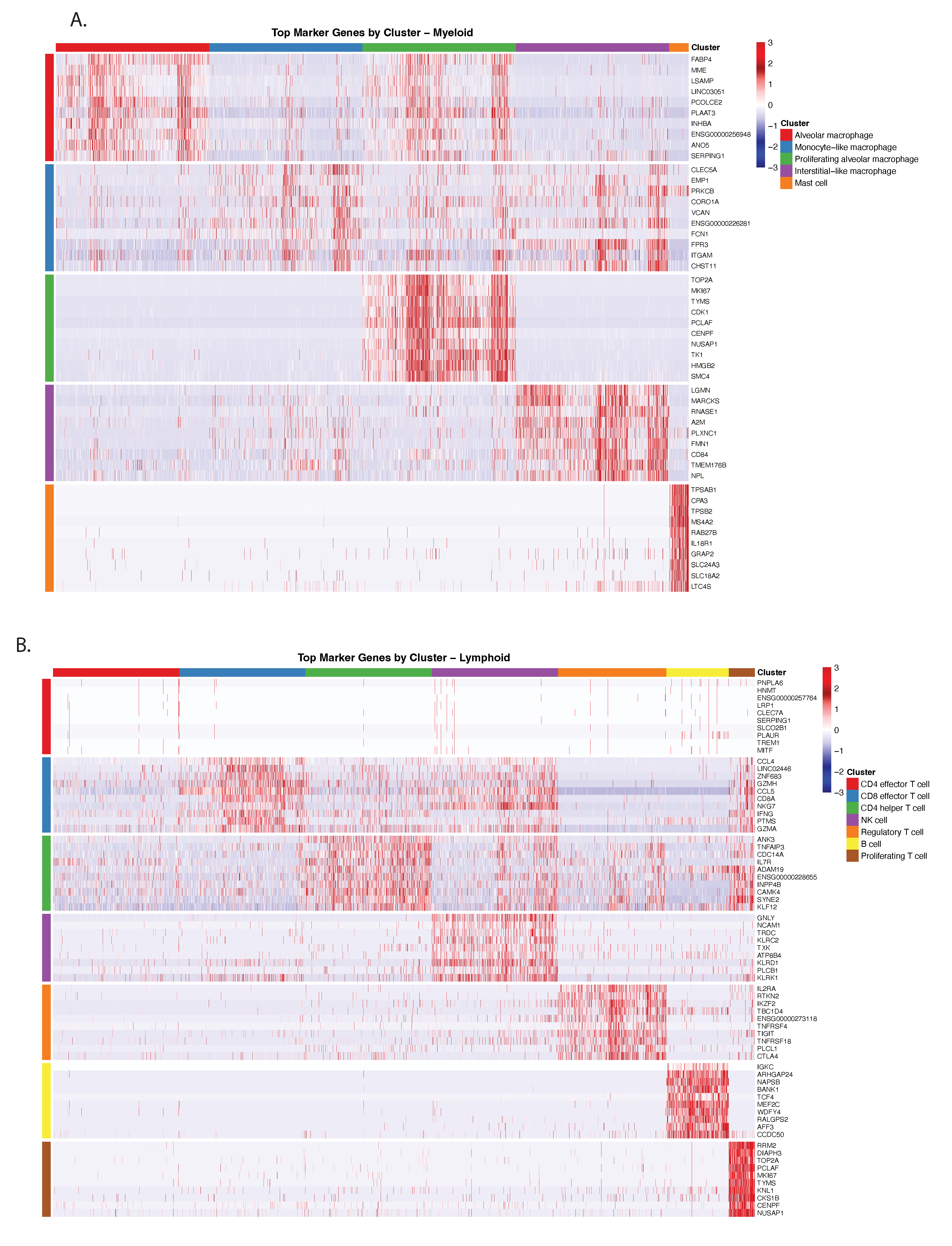

Supplement: S1 Fig — (TIF) [file pone.0347852.s005.tif]

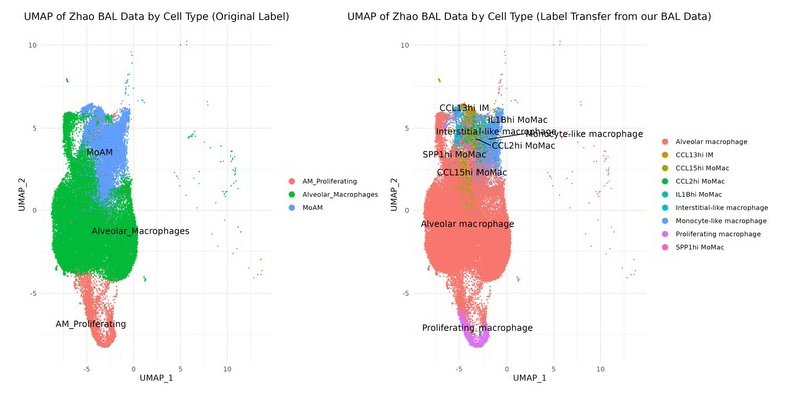

Supplement: S2 Fig — (TIF) [file pone.0347852.s006.tif]

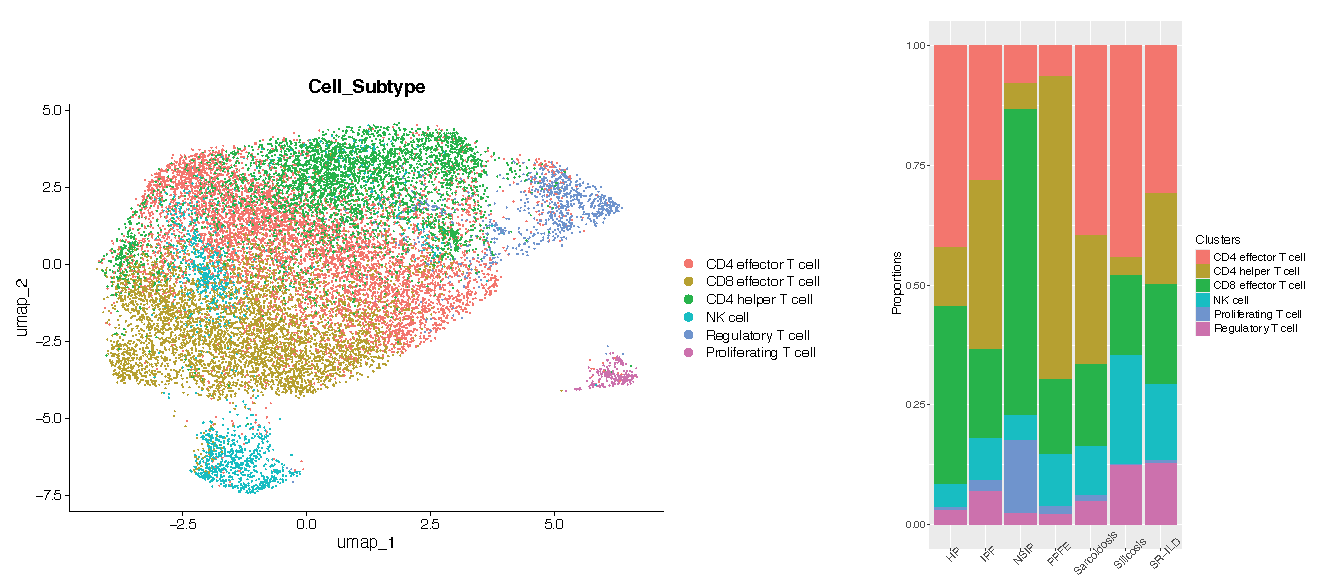

Supplement: S3 Fig — Uniform manifold approximation and projection (UMAP) of T and NK cells isolated from ILD BAL, and cell type proportion analysis of each subcluster (out of all T/NK cells) in each ILD subtype. NK: natural killer. (TIF) [file pone.0347852.s007.tif]

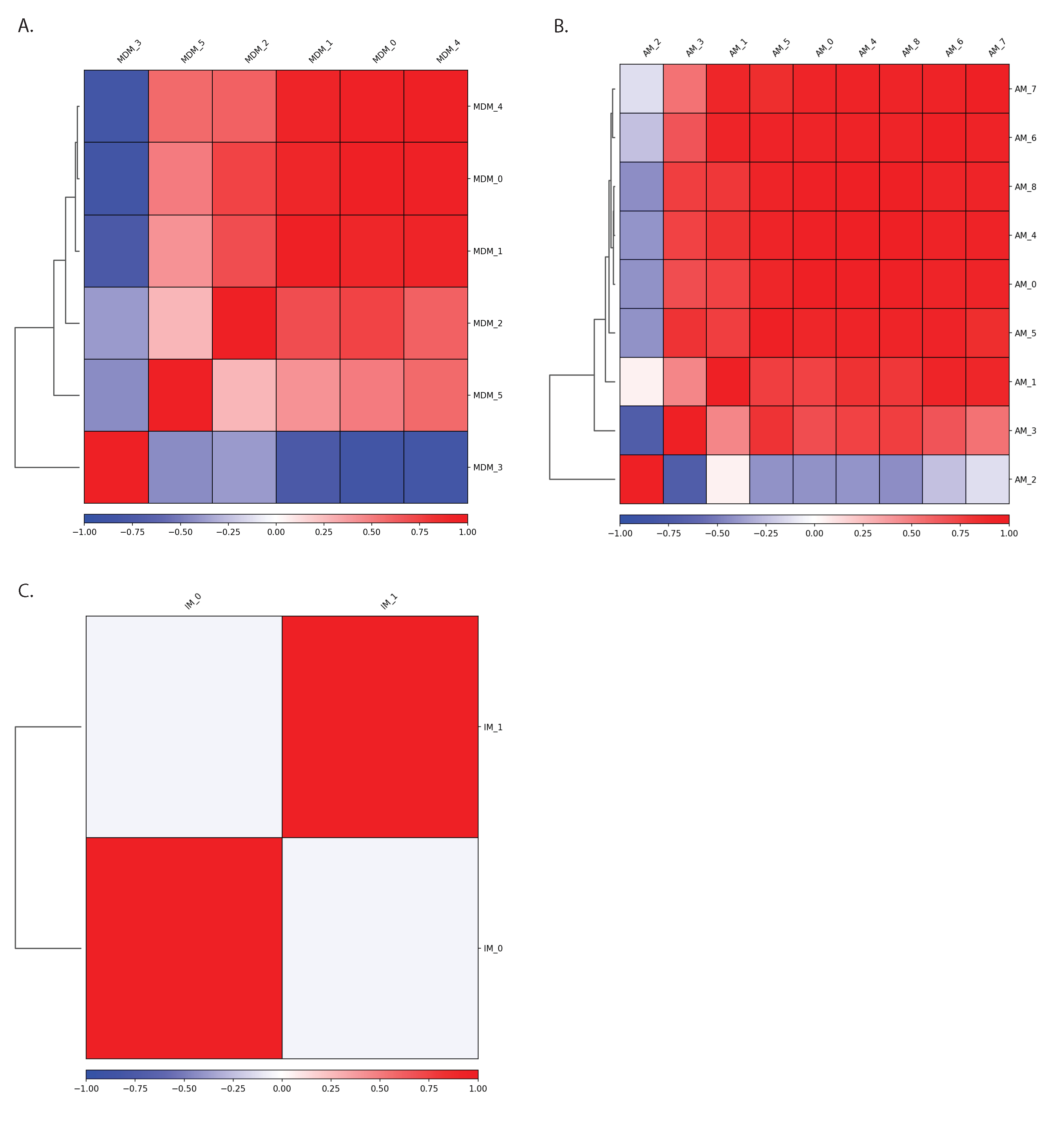

Supplement: S4 Fig — Red indicates positive correlation, blue indicates negative correlation. (TIF) [file pone.0347852.s008.tif]

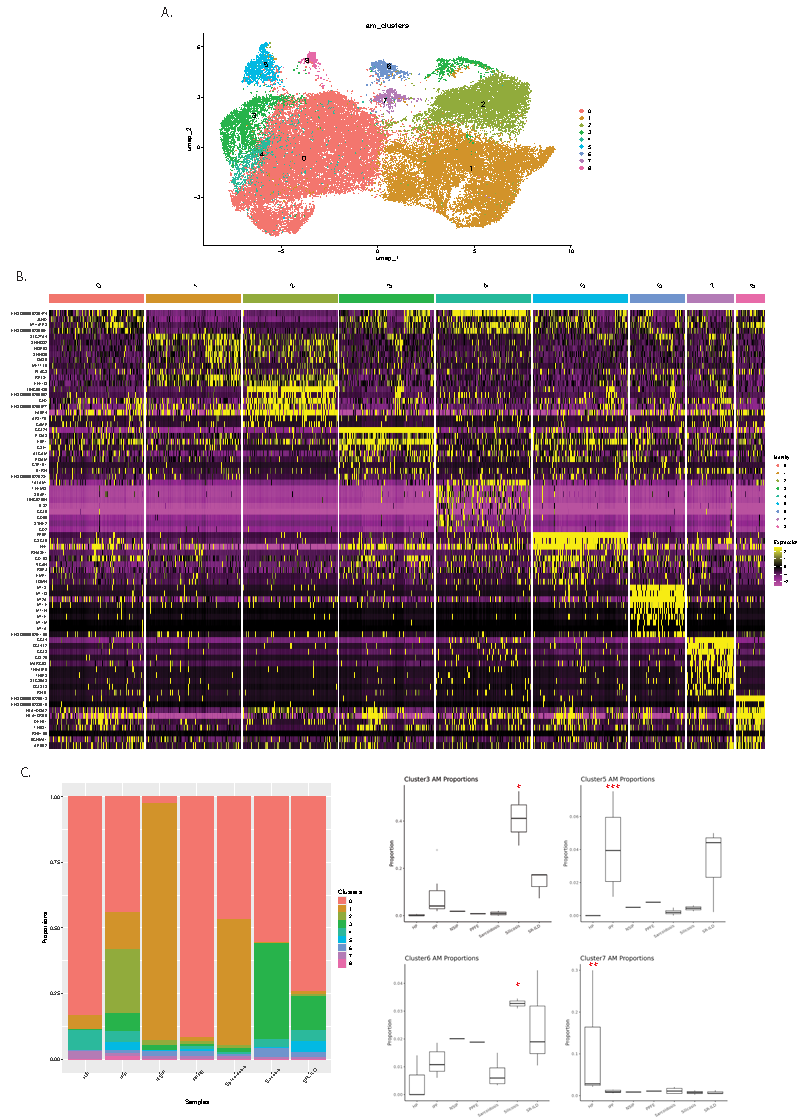

Supplement: S5 Fig — A) Uniform manifold approximation and projection (UMAP) of alveolar macrophage (AM) subclusters isolated from ILD BAL. B) Heatmap of top differentially expressed genes within each AM subcluster. C) Cell type proportion analysis of each alveolar macrophage subcluster (out of all AMs) in each ILD subtype, with significance determined via Wilcoxon rank-sum testing. Only subclusters with significantly different proportions between ILDs are shown. *: p < 0.05, **: p < 0.01, ***: p < 0.001. (TIF) [file pone.0347852.s009.tif]

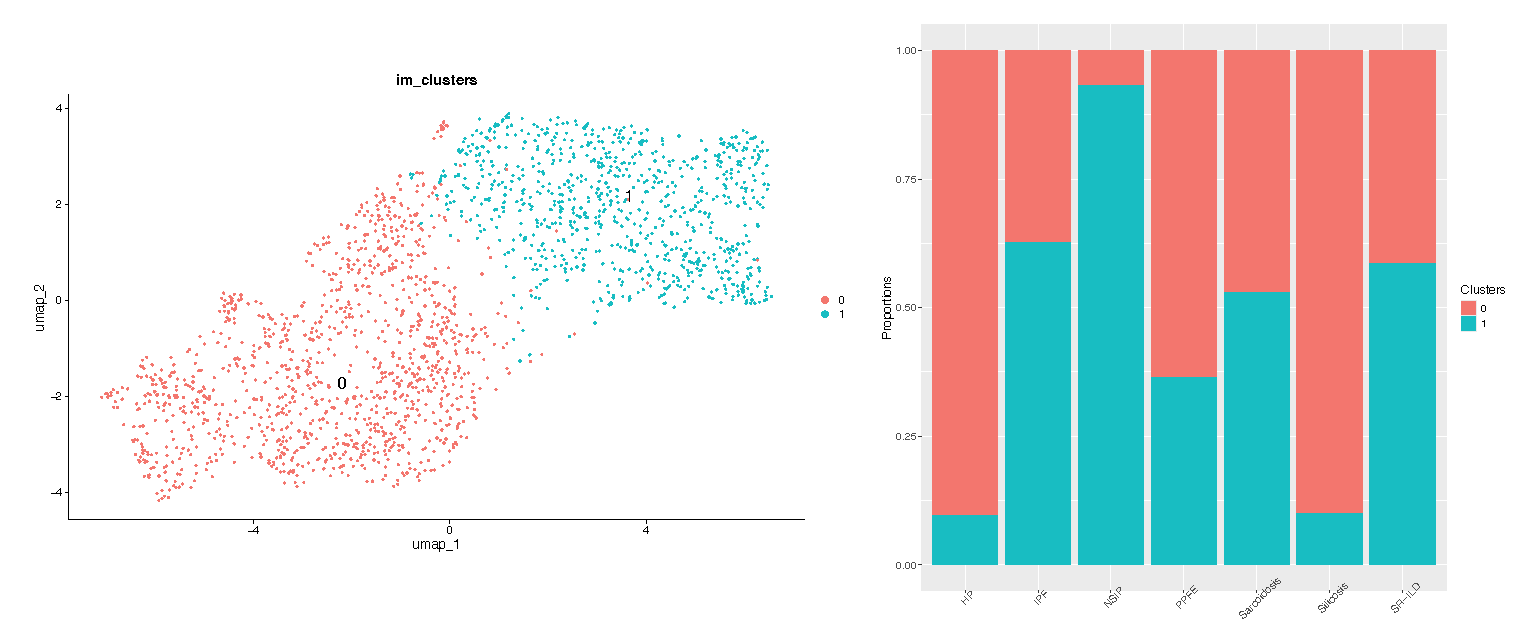

Supplement: S6 Fig — Uniform manifold approximation and projection (UMAP) of interstitial-like macrophage (ILM) subclusters isolated from ILD BAL, and cell type proportion analysis of each ILM subcluster (out of all ILMs) in each ILD subtype. (TIF) [file pone.0347852.s010.tif]
